# Modeling of the Kinetics of Polyoxymethylene Decomposition under Oxidative and Non-Oxidative Conditions

**DOI:** 10.3390/ma14092281

**Published:** 2021-04-28

**Authors:** Tomasz M. Majka, Gabriela Berkowicz-Płatek, Witold Żukowski

**Affiliations:** 1 Department of Chemistry and Technology of Polymers, Cracow University of Technology, Warszawska 24, 31155 Cracow, Poland; tomasz.majka@pk.edu.pl; 2 Department of General and Inorganic Chemistry, Cracow University of Technology, Warszawska 24, 31155 Cracow, Poland; gabriela.berkowicz@pk.edu.pl

**Keywords:** polyoxymethylene, thermal degradation, kinetics, chemical engineering, material engineering

## Abstract

Research on the thermal and thermo-oxidative degradation of polyacetals allows for the development of effective methods of utilization of the waste of these polymers towards the recovery of monomers. For this purpose, in addition to qualitative analysis, it is necessary to understand the mechanisms of chemical reactions accompanying the decomposition process under the influence of temperature. Therefore, in this article, with the experimental results from the thermal analysis of the POM homopolymer of three various stages of life—POM-P—unprocessed sample; POM-R—recycled sample, and POM-O—sample waste—we took steps to determine the basic kinetic parameters using two well-known and commonly used kinetic models: Friedman and Ozawa-Flynn-Wall (OFW). Knowing the values of the course of changes in apparent activation energy as a function of partial mass loss, theoretical curves were fitted to the experimental data. The applied calculation models turned out to be consistent in terms of the nature of the curve changes and similar in terms of *E_a_* in the entire range of mass loss. Both kinetic models showed a very similar course of the *E_a_* curves. The samples that decompose under oxidative conditions obtained the best fit for the reaction of nth order with autocatalysis by product B model and the samples that decompose under inert conditions for the n-dimensional nucleation according to the Avrami–Erofeev model.

## 1. Introduction

Polyoxymethylene (polyacetal, polyformaldehyde, and POM), is a popular thermoplastic engineering polymer due to its good mechanical properties. It has a wide range of applications mainly in the mechanical industry [1,2,3]. However, POM is thermally unstable because its repeating oxymethylene chain units are usually terminated with hemi formal terminals [2,3,4]. POM has a tendency to decompose from these unstable end-chain groups by a chain-cracking process, and in consequence, oxymethylene units are removed from these terminals. This is mainly the case with the homopolymer, because its degree of crystallinity, tensile strength, rigidity, softening, and melting points are increased in comparison with the copolymer.

Various processes of POM degradation have been described in the literature [5,6]. However, they all have one factor in common, degradation, starting on both ends of the polymer chain by split off formaldehyde, some of which is not stopped by ethylene oxide units. This formaldehyde has a characteristic sweet odor during high-temperature processing. Further, the proposed mechanism mainly includes a thermal oxidation process first in the amorphous phase, and next a random chain scission in the crystalline phase connected with hydrolysis and acidolysis [4,5,6,7]. However, it all depends to a great extent on the origin of the polyacetal, the thermal history, the content of additives, and even the heating conditions, as demonstrated in our previous publications [8,9]. 

Therefore, in this publication, we used pure polyacetal (POM-P), recycled polyacetal (POM-R), as well as waste polyacetal (POM-O), and, using mathematical tools, we tried to prove the differences and similarities in the kinetics of the POM degradation process with different life histories in oxidative and inert conditions.

## 2. Background

The starting point for kinetic analysis of the processes taking place in a polymeric material in the glass state is thermo-analytical measurements, i.e., those in which the measured signal, changing in the course of chemical processes, reflects the kinetic nature of the changes taking place [10,11,12,13]. For this purpose, it is necessary to perform several measurements at different sample heating rates [12,14]. As the mechanisms of the degradation processes of polymeric materials are often unknown or very complicated and multi-stepped, two complementary methods have been developed to determine fundamental kinetic parameters: model-free and reaction model fit [15].

Information obtained from establishing model-independent parameters is generally used for quick calculations that provide useful information for model-dependent analysis. The accuracy of isoconversion methods may only be sufficient for single-step reactions as they do not take into account the relationship between the other steps involved. As a result, only one value of apparent activation energy (*E_a_*) is determined. When the pre-exponential coefficient is also determined, it becomes necessary to assume the provision of the function *f* (α) [11,12]. Thus, model-less methods are not suitable for characterizing complex transformations in competitive reactions, where the final result is closely dependent on the heating rate [11,15].

During kinetic analysis, the heated sample is assumed to undergo irreversible reactions according to Equation (1).

(1) $$A\left(s\right)\to B\left(s\right)+B^{\prime }\left(g\right)↑$$

The reaction described in Equation (1) also describes the following Equation (2) as a function of known parameters.

(2) $$\frac{dp}{dt}=U\left(t,\;T,\;e,\;p\right)$$

where *p* is the concentration of products; *e*—the initial concentration of the reactants; *t*—time; *T*—temperature.

The right side of this expression is the so-called conversion function that can be written as the product of two separate functions (Equation (3)):

(3) $$U\left(t,\;T,\;e,\;p\right)=k\left[T\left(t\right)\right]f\left(e,p\right)$$

The separation of variables in this equation is possible only for single-step reactions. This is related to the reduction of the term *f*(*e*,*p*) to *f*(*e*). The quantities *e* and *p* are interdependent:

*e* = 1 − *α*, *p* = *α* (4)

where the degree of conversion, α, is defined according to Equation (5):

(5) $$\alpha =\frac{m_{0}-m}{m_{0}-m_{\infty }}$$

where *m*_0_—initial sample mass; *m*—the mass of the current sample; *m*_∞_—final mass.

If multi-stage processes are considered, obtaining an analytical solution of Equation (3) becomes impossible due to the appearance of a system of differential equations for which variables cannot be separated [14,16]. The term *k*(*T*) in Equation (6) defines the temperature dependence of the reaction rate constant, described by the classical Arrhenius equation in the active collision theory.

(6) $$k\left(T\right)=Aexp\left(\frac{-E_{a}}{RT}\right)$$

where *R*—gas constant.

Substituting Equation (6) into Equation (2), we can obtain the reaction rate equations (Equations (7) and (8)):

(7) $$\frac{dp}{dt}=Aexp\left(\frac{-E_{a}}{RT}\right)f\left(e,p\right)$$

(8) $$\nu_{j}=A_{j}exp\left(\frac{-E_{aj}}{RT}\right)f_{j}\left(e_{j},p_{j}\right)$$

where ν—the reaction rate.

Equation (8) is used for *j*-step processes where each elemental reaction is described by a separate kinetic equation. The more the concentrations of the reactants increase, the greater the number of reactions of a given reagent in a product. Additionally, the concentrations of the reactants decrease with the increase in the reactions in which this reagent is a substrate [11].

In the course of research on the geometry of reaction surfaces, a number of kinetic models were suggested. The selected forms of functions, as well as the kinetic models of reactions, are summarized in Table 1.

In order to correctly define the kinetic model of the degradation reaction, several basic assumptions were made:

- The reaction is the sum of separate steps in chemical reactions, characterized by a constant value of activation energy and is a mathematical expression of a kinetic model;
- Reactions can take place independently, concurrently, or sequentially;
- The reactions take place in an irreversible manner during the thermal degradation of polymers, because the volatile degradation products formed during the thermogravimetric analysis are continuously removed from the apparatus’ furnace chamber.

After presenting the described assumptions using the Friedman and Ozawa-Flynn-Wall (OFW) isoconversion equations, it is worth introducing the definition of the heating rate (*β*) into Equation (7), which also replaces the *p* by α.

Finally, Equation (9) is obtained as:

(9) $$\frac{d\alpha }{dT}=\frac{A}{\beta }exp\left(\frac{-E_{a}}{RT}\right)f\left(\alpha \right)$$

Friedman [18,19] proposed a relationship between the logarithm of the transformation rate $\left(d\alpha /dt\right)$ and the reciprocal of temperature 1/*T*, providing the possibility to calculate the activation energy value (Equation (10)):

(10) $$\ln\left(\beta \frac{d\alpha }{dT}\right)=\ln A+\ln f\left(\alpha \right)-\frac{E_{a}}{RT}$$

On the basis of the shown linear dependence, a series of straight lines is drawn in the ln [*β*·(*d*α/*dT*)] system from the reciprocal of the absolute temperature. Each of these lines corresponds to a specific conversion rate, and the apparent activation energy is read as its directional factor.

The second isoconversion method introduced by Ozawa, Flynn, and Wall (OFW), also for determining activation energy, is based on the following considerations. The principle of the calculations is analogous to that presented in the Friedman method, but here, the dependence on the heating rate factor is introduced, and Equation (7) is transformed into the form of the expression presented in Equation (11).

(11) $$\ln\beta =-1.0516\frac{E_{a}}{RT}-5.3305$$

If the above equation is plotted in the coordinate system ln (*β*)—ordinate axis and 1/*T*—abscissa axis, then for a series of measurements made at different heating rates, for a given conversion degree α, rectilinear graphs are obtained with a slope of 1.052 *E*/*R*. The temperature *T_jk_* is the temperature at which the degree of conversion α*_k_* is achieved at the heating rate *β_i_* (the indexes *i*, *j*, and *k* denote the set of experiments performed under different heating rates) [16,20,21].

The optimization of the theoretical curve fit to the experimental data is based on the numerical Runge-Kutta computation algorithm, which is part of the modified Marquardt iteration procedure [22,23,24]. It allows the systems of differential equations describing the various kinetic models presented in Table 1, i.e., the relationships among independent, subsequent, parallel, and competitive reactions, to be solved. The general symbols used for these types of reactions are shown in Table 2.

## 3. Materials and Methods 

### 3.1. Materials

POM-P obtained from DuPont Poland (Delrin^®^ 100 NC010, Warsaw, Poland) was a pure sample (reference sample) of a high-viscosity acetal homopolymer with no thermal processing history.

Using a Thermo Scientific HAAKE Rheomex PTW 16/25 XL (Thermo Fisher, Karlsruhe, Germany) twin-screw extruder (L/D factor 25), we obtained a sample of recycled polyoxymethylene (POM-R) according to the following processing conditions:

- Drying temperature: 80 °C;
- Drying time: 4 h;
- Temperature of feed zone: 190 °C;
- First zone temperature: 200 °C;
- Second zone temperature: 200 °C;
- Third zone temperature: 210 °C;
- Forth zone temperature: 210 °C;
- Extrusion zone temperature: 220 °C;
- Die temperature: 220 °C;
- Screw speed: 120 rpm.

The waste acetal (POM-O) was a cut sample of gear wheel (Cutter for plastics, Rapid 2B, Cracow, Poland) Atlanta 22 10 018 (ATLANTA Antriebssysteme, Bietigheim-Bissingen, Germany) purchased at the Czescimaszyn24 store (Babice, Poland).

### 3.2. Differential Scanning Calorimetry (DSC)

Changes in thermal properties were determined by differential scanning calorimetry (DSC). For this purpose, a Mettler Toledo DSC823^e^ calorimeter (Mettler-Toledo Sp. z o.o., Warsaw, Poland) was used. Calorimetric measurements were carried out in an oxidizing atmosphere. The calorimeter was calibrated with indium and zinc. The samples weighing ~5 mg were placed in an aluminum pan, and sealed in a press. The measurements were made in accordance with two heating scans, which also allowed for the assessment of thermal properties after annealing:

- First heating (I) from 25 to 200 °C with a heating rate of 10 °C/min;
- Cooling from 200 to 25 °C with cooling rate 10 °C/min;
- Second heating (II) from 25 to 200 °C with a heating rate of 10 °C/min.

The degree of crystallinity (*X_c_*) of POM was determined by using the following equation:

(12) $$X_{C}=\frac{\Delta H_{m}}{\Delta H_{m}^{0}}\cdot 100\%$$

where Δ*H_m_* is the measured heat of fusion of POM sample, and Δ*H_m_*^0^ is the heat of fusion of 100% crystalline POM.

### 3.3. Thermogravimetric Analysis

The thermogravimetric analysis was performed under synthetic air as well as inert atmosphere (Ar), using a Netzsch TG 209 F1 Libra thermogravimetric analyzer (Netzsch Group, Warsaw, Poland). The measurements were conducted in the temperature range from 20 to 600 °C at a heating rate of 10, 15, and 20 °C/min, in the synthetic air atmosphere with an airflow rate of 15 cm^3^/min, and also in an inert atmosphere at a heating rate of 10 °C/min with a gas flow rate of 15 cm^3^/min. Samples of ~5 mg mass were placed in open corundum pans. The raw data were converted into ASCII files.

### 3.4. Kinetic Modelling of Thermo-Oxidative Degradation Process 

Kinetic analyses were carried out using in-house software and a Netzsch Thermokinetic Software (v. 99/10) (Netzsch Group, Warsaw, Poland). These analyses included the determination of apparent activation energy and the lg(A/s^−1^) factor by the Friedman and OWF method. Based on the obtained data, simulations of the best fit of kinetic models to experimental data were performed. Then, knowing the kinetic model for which the factor F-test is equal to 1 and determining the remaining theoretical kinetic parameters of the degradation process, it was possible to predict the thermo-oxidative degradation rate in the temperature range 300–370 °C. The temperature range was dictated by the fact that in our previous publication [8,9], we suggested that the thermal degradation of POM should be carried out at least at 400 °C and the combustion process could be performed at a temperature as low as 350 °C. When performing computer calculations, a procedure was used that was developed taking into account the examples given in the literature [13,15,16,22,23,24,25,26,27,28]. 

## 4. Results and Discussion

### 4.1. Differential Scanning Calorimetry (DSC)

**Figure 1:**
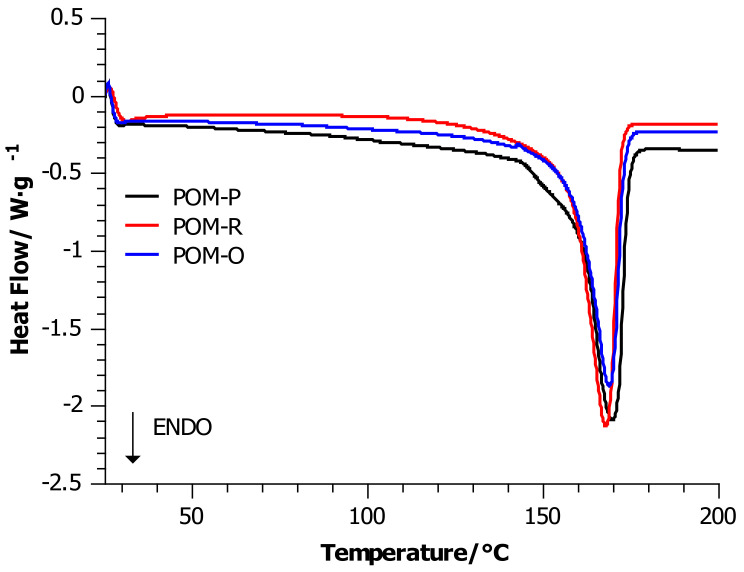
DSC heating curves for POM-P, POM-R, and POM-O samples.

**Figure 2:**
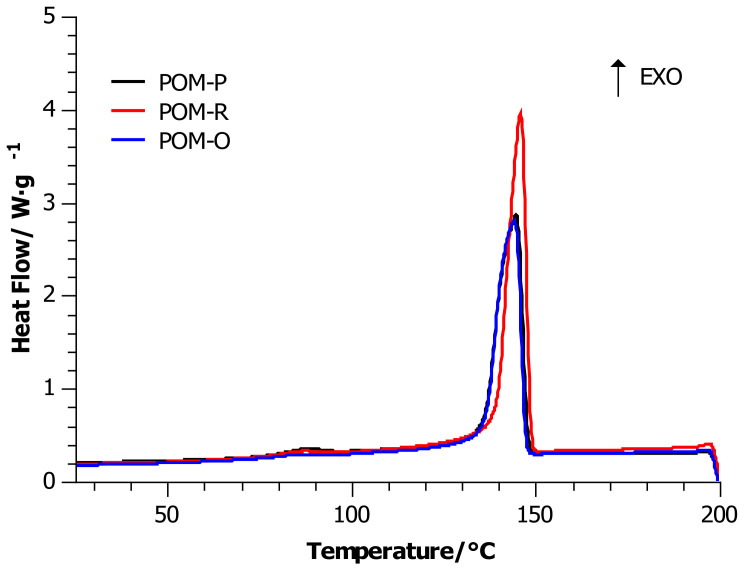
DSC cooling curves for POM-P, POM-R, and POM-O samples.

Figure 1 shows the curves recorded during heating at 10 °C/min, and Figure 2 shows the curves noted during cooling at 10 °C/min. Based on the analysis of DSC curves, the glass transition temperature, the maxima corresponding to the melting points of the crystalline phases present in the samples, changes in the melting enthalpy, changes in specific heat, and changes in the crystallization temperature were determined. The degree of crystallinity was determined according to the procedure published in [29,30].

The above-mentioned properties are summarized in Table 3. All curves were characterized by the dominant phenomenon of energy absorption by the system. Throughout the entire study cycle, the samples showed an endothermic effect. It was observed that samples of pure POM and its waste were characterized by an increased endothermic effect. In the cases of the recycled (processed) sample, it remained slightly smaller, even after being heated twice.

In order to calculate the degree of crystallinity, the value of 326.3 J/g was assumed as ∆*H* of 100% crystalline POM [31]. The calculations showed that material recycling slightly increased the degree of crystallinity in relation to the unprocessed sample. On the other hand, multiple processing and long-term use reduced the degree of crystallinity to 43%. With regard to the material, its potential and constant exposure to atmospheric, mechanical, thermal, or environmental factors is minimal. Observing these slight changes in the values of the degree of crystallinity, one may be tempted to say that POM is an extremely durable polymer for operational conditions.

The change in POM-specific heat accompanying melting process decreased after its processing by nearly 16%. A smaller decrease was noted for the waste sample (POM-O, 14%). This means that both the processing and use of the POM detail facilitates the melting of the material, i.e., the easy transition from a glassy to a plastic state, because less energy must be supplied to the system to reach the transition point between these states. This might be due to, inter alia, a reduction in the content of crystalline phases caused by high-temperature processing combined with rapid cooling of the system. The samples showed a comparable change in the slope of the base curves. Another change observed was the melting of the samples. All the curves showed a single characteristic peak, although accentuated curve inflection was observed for the POM-P reference sample at 145 °C. This might indicate the existence of a small amount of unstable crystalline phase in pure polyoxymethylene, which disappears after processing the sample (POM-R). In the case of the POM-O sample, the presence of additives (dyes and stabilizers) probably emphasized the side peaks reflecting the overlapping of other energy effects in the range of 130–170 °C. The presence of a broad and flat peak in this temperature range can be interpreted as the melting process of the less stable phase, which in this case had an insignificant share. Unfortunately, neither the processing nor the use of POM influenced the melting point of the produced stable crystalline phase observed during both the first and the second heating, and slight shifts were not within the apparatus error.

DSC cooling curves at 10 °C/min showed (Figure 2) a similar slope of the baseline (especially POM-P and POM-O) and one exothermic peak in the range of 160–130 °C. All materials have one maximum point, designated as crystallization temperature *T_cr_*. Crystallization temperature values for POM-P, POM-O, and POM-R samples differed by 2 °C. The angle of inclination of the ascending lines of the exothermic peak in relation to the baseline allowed us to conclude that the faster stage of the crystallization process for all tested samples was the one just before reaching the maximum intensity of energy release in the crystallization process. Nevertheless, the slope of the baseline for the POM-R sample material was much softer than for the corresponding reference sample.

### 4.2. Thermogravimetric Analysis

**Figure 3:**
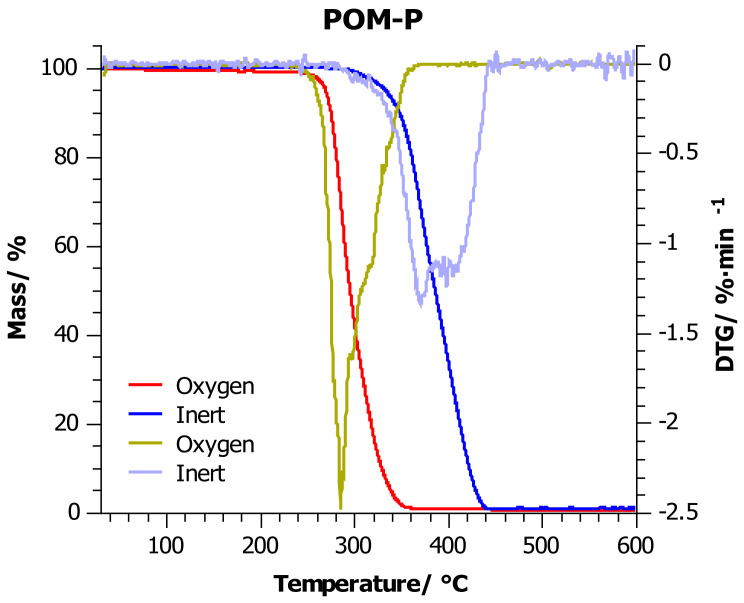
TG thermograms for POM-P sample: red line—investigations carried out in oxygen atmosphere; blue line—investigations carried out in the inert atmosphere, at a heating rate of 10 °C/min.

**Figure 4:**
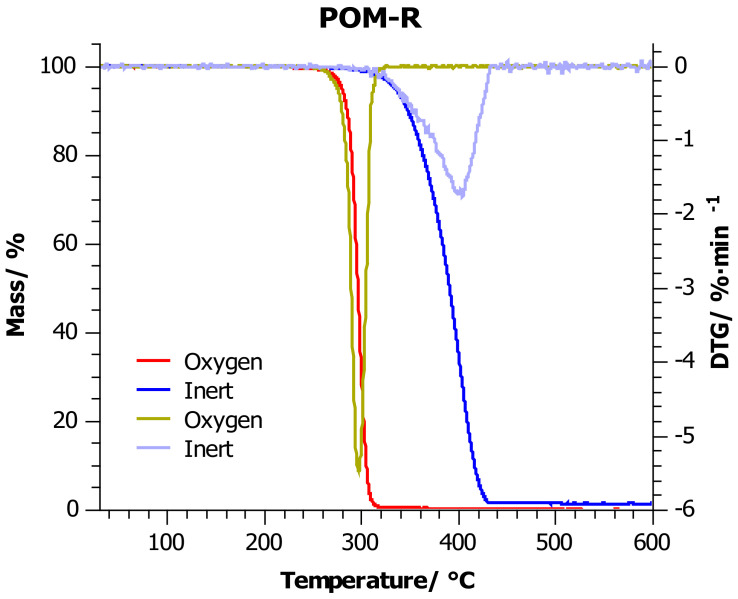
TG thermograms for POM-R sample: red line—investigations carried out in oxygen atmosphere; blue line—investigations carried out in the inert atmosphere, at a heating rate of 10 °C/min.

**Figure 5:**
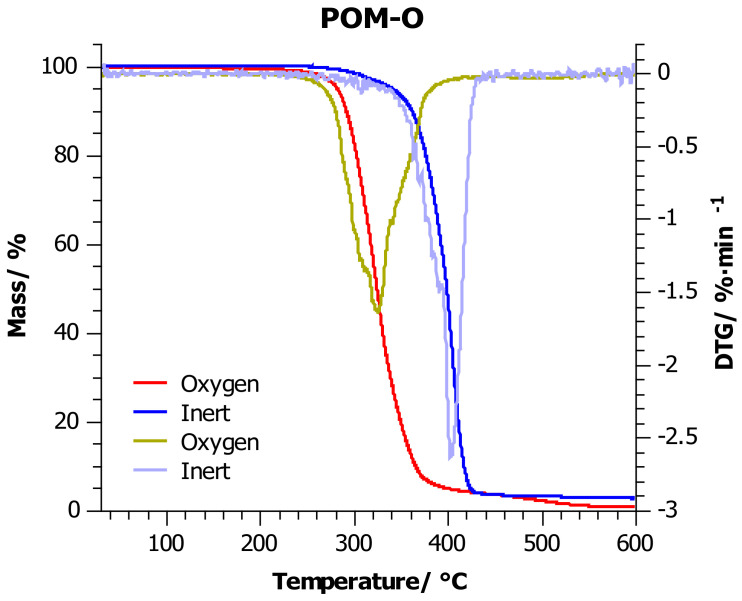
TG thermograms for POM-O sample: red line—investigations carried out in oxygen atmosphere; blue line—investigations carried out in the inert atmosphere, at a heating rate of 10 °C/min.

Figure 3, Figure 4 and Figure 5 show the thermogravimetric curves for POM-P, POM-R, and POM-O samples obtained during the measurement in an oxidizing atmosphere (red line) and in an inert atmosphere (blue line). Additionally, Table 4 lists the most important thermogravimetric indicators in the graphs, which facilitate the analysis of the collected data.

Considering the measurements carried out in the inert atmosphere, it was observed that in the range of 25–370 °C, the POM-P and POM-R samples had a very similar thermogram. Above this temperature, the reference sample (POM-P) was less thermally stable. The reason for this was that the sample reached the point of maximum weight loss just at 370 °C, while the POM-R sample reached this point at 34 °C higher.

Testing in an inert atmosphere makes it possible to compare the effect of temperature itself on mass loss (decomposition/thermal stability of substances), and thus to assess and compare the behavior of materials at high temperatures with different chemical structures or compositions. In the discussed case, the unprocessed sample was more susceptible to temperatures above 370 °C than the repeatedly processed sample (POM-R). The reason for this is the different thermal histories of the compared samples, which is confirmed by the DSC test. 

As a result of multiple processing, the POM-R polymer chain was already subjected to state changes, by inducing segmental movements within the macromolecule and stopping them, or by creating crystalline phases in greater amounts than can be present in the reference sample (POM-P). Thus, the processed sample became more resistant to the linearly rising temperature and more than the reference sample. Nevertheless, the reference sample degraded more slowly than the POM-R sample, which indicated a shift of the endset point of the TG curve towards higher temperatures. The residue after the measurement was 1% (600 °C) in both cases, which may indicate that the same point after decomposition was finally reached. The study carried out for the waste POM showed that this material was characterized by much better thermal stability at temperatures up to 400 °C (TG indicators higher by nearly 10 °C). Only after exceeding the point of maximum weight loss, the endset on the TG curve was reached quickly and, consequently, over 3% of the residue at 600 °C. Such a result may prove that the waste sample was enriched with components that did not decompose up to 600 °C (there were at least 2% of them), and they are responsible for the good thermal stability of the material at lower temperatures. Nevertheless, in all the cases discussed, one major stage of decomposition was noted.

In the case of measurements carried out in an oxidizing environment, as before, the POM-P and POM-R samples showed similar thermal stability at lower temperatures, which began to differ only after the *T*_50%_ point was exceeded. A deeper analysis of the endset on the TG curve and the curve itself in the temperature range 300–400 °C showed faster residue burning in the POM-R sample. Polymer recycled (POM-R), definitely in the presence of oxygen, was more susceptible to combustion than the reference sample (POM-P). In such an environment, the degree of degradation initiated at lower temperatures played a major role, which in turn led to the faster decomposition of the polymer at much higher temperatures. The influence of the oxidizing environment also caused a reduction in all indices by nearly 60 °C for *T*_5%_–*T*_20%_, and even 100 °C for *T*_50%_ and *T_max_*, compared to the previously discussed results. Differences in the residual values between the two samples at the end temperatures of the heating program suggest that different degradation mechanisms are involved. Nevertheless, such a low residue content (0.6% POM-P and 1% POM-R and POM-O) indicates that good flammability of these samples can be expected. The waste sample (POM-O) was also characterized by the highest thermogravimetric indicators (Table 4); however, under oxidizing conditions, two decomposition stages could be observed for this sample: the first in the range up to 390 °C, which was the main polymer decomposition, and the second from 390 to 560 °C, which was the process of afterburning the carbon to a residue of 1%. It can be concluded that the residues formed after heating the POM-O sample in an inert environment (which may be responsible for the material stabilization effect at lower temperatures) decompose at temperatures of 390–560 °C in an oxidizing environment during the afterburning of the resulting residue.

### 4.3. Kinetic Modelling of Thermo-Oxidative Degradation Process

**Figure A1:**
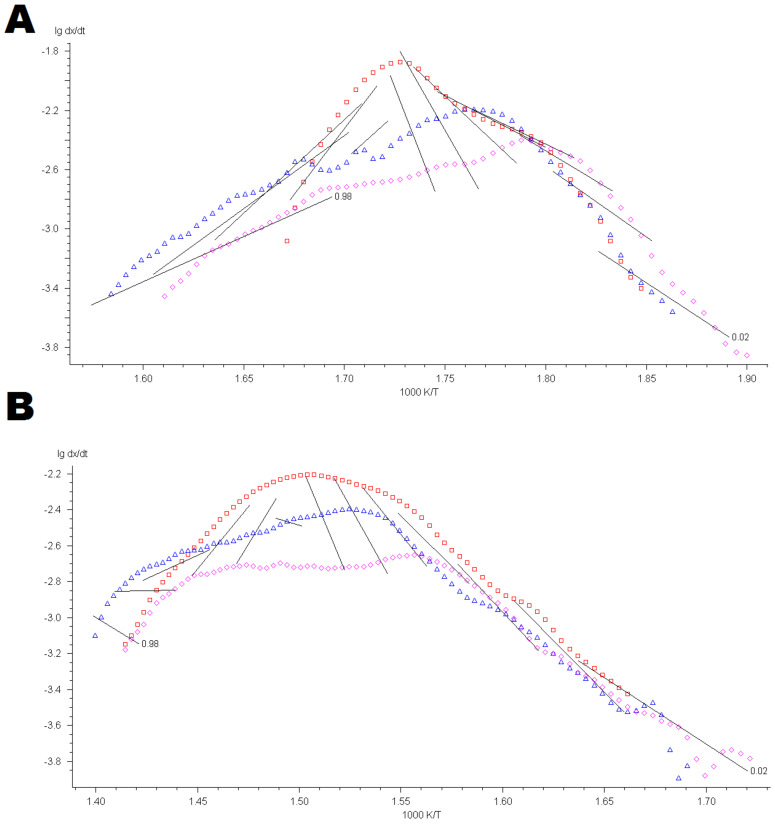
Friedman POM-P analysis plot lg (reaction rate) vs. 1000 K/T: (**A**) sample investigated in an oxygen atmosphere; (**B**) sample investigated in an inert atmosphere. Lines are drawn through isoconversion points for the measurement with 10 (◊), 15 (∆), and 20 (□) °C/min heating rates.

**Figure A2:**
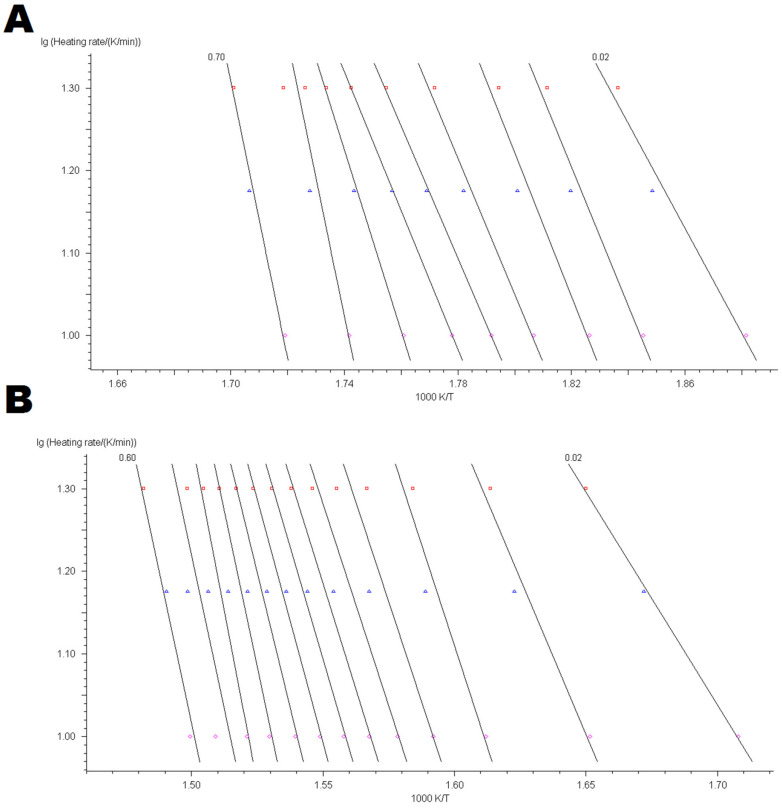
Ozawa-Flynn-Wall POM-P analysis plot lg (heating rate) vs. 1000 K/T: (**A**) sample investigated in an oxygen atmosphere; (**B**) sample investigated in an inert atmosphere. Lines are drawn through isoconversion points for the measurement with 10 (◊), 15 (∆), and 20 (□) °C/min heating rates.

**Figure A3:**
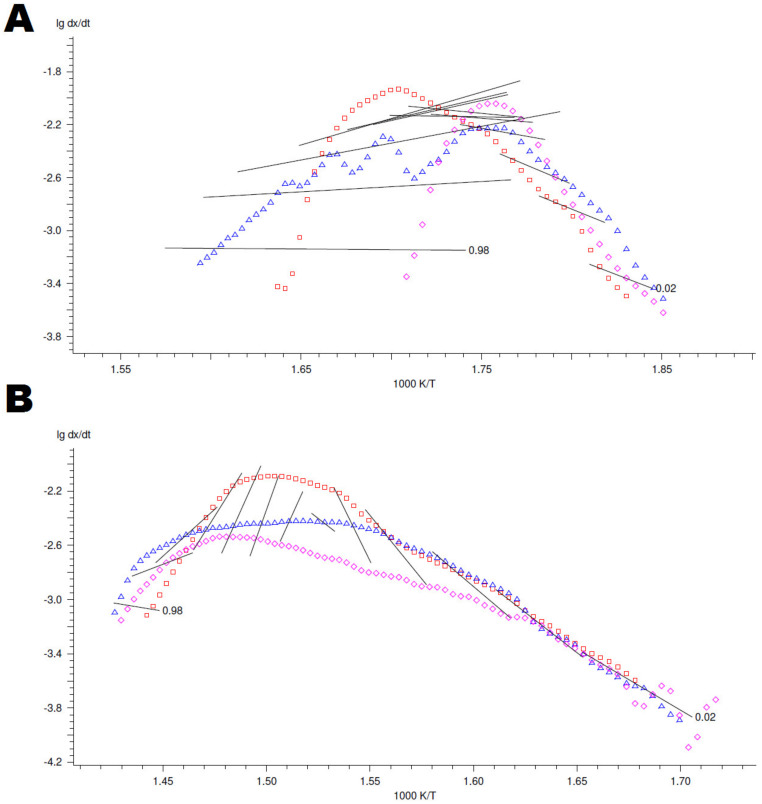
Friedman POM-R analysis plot lg (reaction rate) vs. 1000 K/T: (**A**) sample investigated in an oxygen atmosphere; (**B**) sample investigated in an inert atmosphere. Lines are drawn through isoconversion points for the measurement with 10 (◊), 15 (∆), and 20 (□) °C/min heating rates.

**Figure A4:**
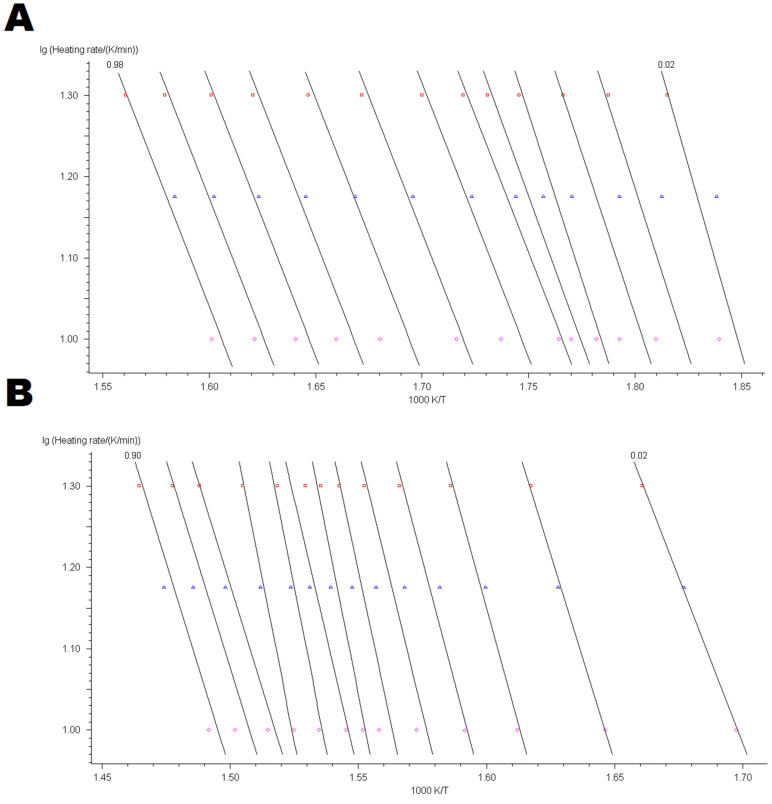
Ozawa-Flynn-Wall POM-R analysis plot lg (heating rate) vs. 1000 K/T: (**A**) sample investigated in an oxygen atmosphere; (**B**) sample investigated in an inert atmosphere. Lines are drawn through isoconversion points for the measurement with 10 (◊), 15 (∆), and 20 (□) °C/min heating rates.

**Figure A5:**
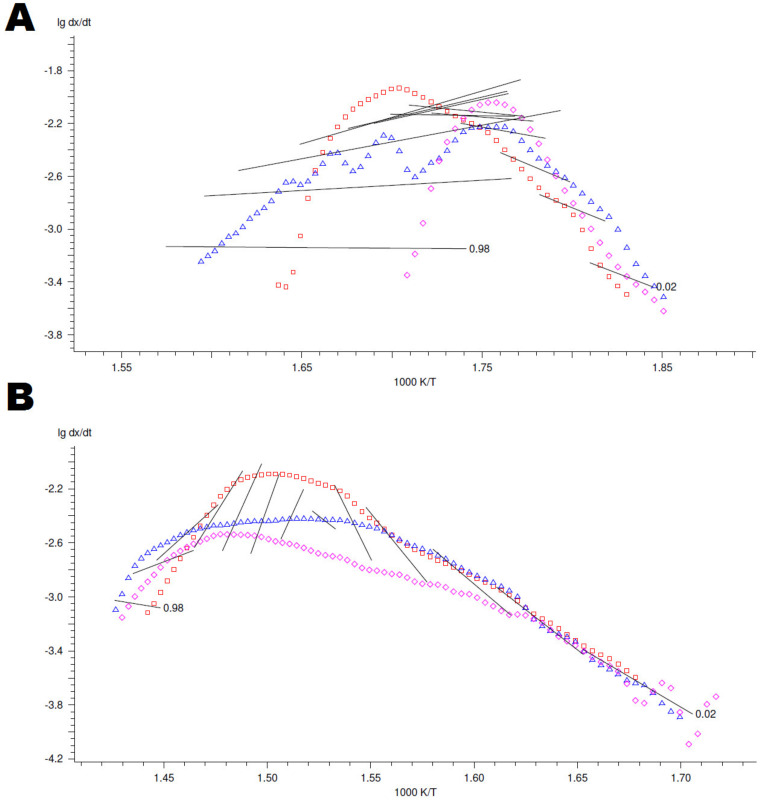
Friedman POM-O analysis plot lg (reaction rate) vs. 1000 K/T: (**A**) sample investigated in an oxygen atmosphere; (**B**) sample investigated in an inert atmosphere. Lines are drawn through isoconversion points for the measurement with 10 (◊), 15 (∆), and 20 (□) °C/min heating rates.

**Figure A6:**
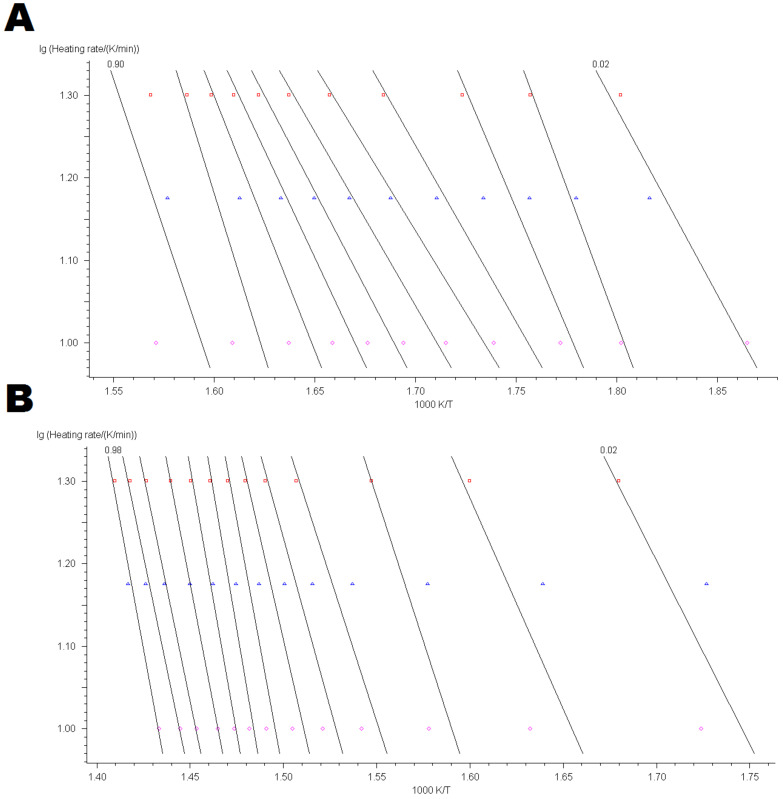
Ozawa-Flynn-Wall POM-O analysis plot lg (heating rate) vs. 1000 K/T: (**A**) sample investigated in an oxygen atmosphere; (**B**) sample investigated in an inert atmosphere. Lines are drawn through isoconversion points for the measurement with 10 (◊), 15 (∆), and 20 (□) °C/min heating rates.

In order to obtain significant information on the mechanism of the degradation process, determination of the reaction order, reaction rate constant, apparent activation energy, and the pre-exponential coefficient, kinetic studies of the POM-P, POM-R, and POM-O thermal degradation process was carried out using the thermogravimetric method. A set of three signals with the following temperature domains was introduced as thermogravimetric input data: *β* = 10 °C/min, 20–600 °C; *β* = 15 °C/min, 20–600 °C; *β* = 20 °C/min, 20–600 °C. Figure A1, Figure A2, Figure A3, Figure A4, Figure A5 and Figure A6 (Appendix A) show the results of the analysis of the kinetic degradation of POM-P, POM-R, and POM-O samples in an oxidizing (A) OX and inert (B) IN atmosphere for the Friedman and OFW models. The pink color corresponds to the heating rate *β* = 10 °C/min, blue *β* = 15 °C/min, and red *β* = 20 °C/min.

As shown by the curves for the samples tested under oxidizing conditions (Appendix A: Figure A1A, Figure A3A and Figure A5A), all three POM thermogravimetric curves showed a course trend characterized by noticeable grades. The comparison of two different isoconversion methods (Friedman and OWF—Appendix A) proved their differences and imperfections when applied to numerous studies of the degradation of polymeric materials [25,26,27,28]. On the basis of the obtained curves, the values of apparent activation energy (*E_a_*) in the entire range of mass loss were determined using both kinetic models.

**Figure 6:**
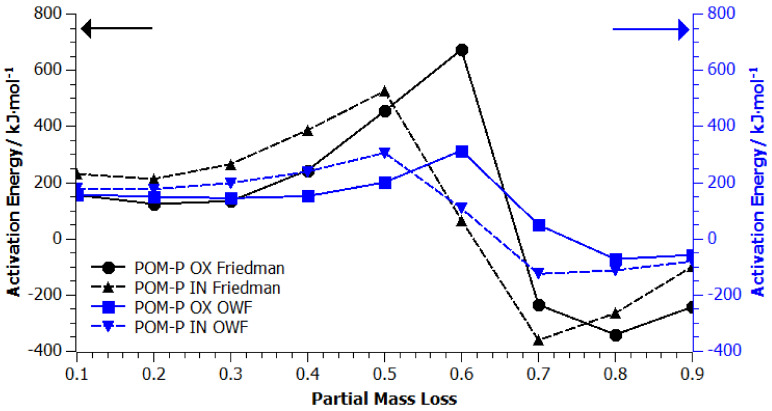
Apparent activation energy vs. partial mass loss found from the slope of isoconversion lines from Friedman (black lines) and OWF (blue lines) analyses of POM-P plots, where OX stands for tests carried out in an oxidizing atmosphere and IN in an inert atmosphere.

**Figure 7:**
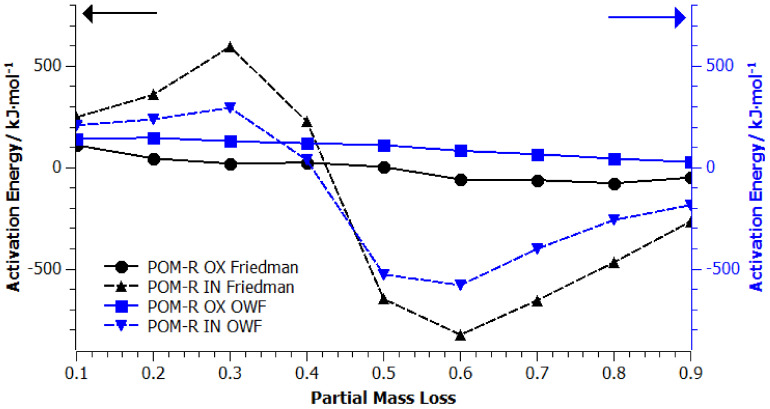
Apparent activation energy vs. partial mass loss found from the slope of isoconversion lines from Friedman (black lines) and OWF (blue lines) analyses of POM-R plots, where OX stands for tests carried out in an oxidizing atmosphere and IN in an inert atmosphere.

**Figure 8:**
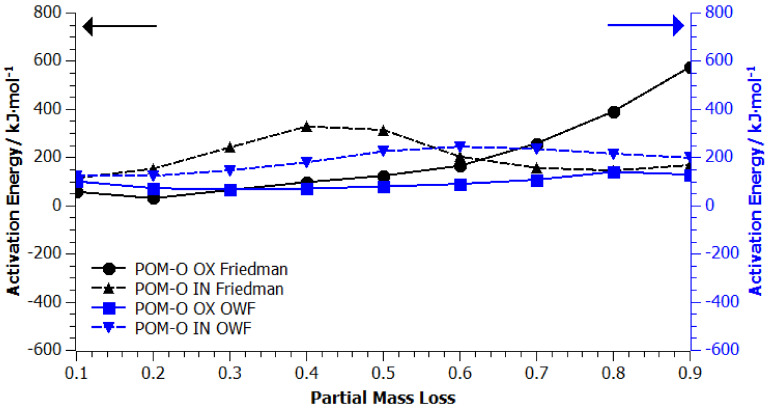
Apparent activation energy vs. partial mass loss found from the slope of isoconversion lines from Friedman (black lines) and OWF (blue lines) analyses of POM-O plots, where OX stands for tests carried out in an oxidizing atmosphere and IN in an inert atmosphere.

The course of the curves shown in Figure 6, Figure 7 and Figure 8 confirmed that the decomposition of homopolyacetals can be a single or multi-stage process depending on their origin and thermal history, which may result, among other factors, from only univariate or invariant apparent activation energy as the reaction progresses. Following the literature data, it would be expected that the activation energy for the homopolymer should be in the range of 40–160 kJ/mol [3,4].

In the case of the graph of changes in apparent activation energy as a function of the partial mass loss of the POM-P sample, a similar trend of changes in activation energy was observed in the range of 0.4–0.8 weight loss (regardless of the nature of the atmosphere in which TG measurements were carried out and the calculation model used). However, the intensity of the apparent activation energy changes in the considered range seemed to be much greater for the results calculated by the Friedman method. The obtained values of apparent activation energy (500–650 kJ/mol—Friedman model; 250–300 kJ/mol—OWF model) significantly exceeded those obtained in the literature. This is due to the fact that the POM-P sample did not have any thermal history; hence, it was characterized by a higher energy barrier, which had to be exceeded to lead to decomposition both under oxidizing and inert conditions. On the other hand, the data presented in the literature came from the POM sample, which, like POM composites, already had a certain thermal history. According to the literature, an increase in the activation energy can be attributed to the higher degree of crystallinity of POM [32,33] manifested by the better arrangement of polymer chains even in the molten state. More crystalline polymers require higher energy inputs to overcome their inter- and intramolecular forces, and the activation energy barrier is at higher level [32,33].

On the other hand, in the case of the recycled sample (POM-R), the nature of changes in apparent activation energy was dictated by the conditions for conducting thermogravimetric measurements. The applied calculation models turned out to be consistent in terms of the nature of the curve changes and similar in terms of *E_a_* in the entire range of mass loss. It is worth noting that the trend of *E_a_* changes in the oxidizing medium (OX) with the progress of the reaction corresponded to the trend presented in the cited literature [3,4,32]. Meanwhile, the degradation of POM-R carried out in an inert atmosphere followed a different mechanism, which covered almost the entire measuring range. Its maximum was shifted towards lower temperatures and also lower values of mass loss. The only factor that remained unchanged was the height of the energy barrier. The presented observations can be summed up by the fact that POM with a single thermal history decomposes quite easily under oxidative conditions, while the same sample under neutral conditions has a high energy barrier (like the POM-P sample) with the maximum shifted towards lower temperatures. 

Multiple processing of polyacetal (POM-O) and exposure of the polymer to other external factors (mechanical stress, UV radiation, etc.) resulted in a significant reduction in the apparent activation energy in the entire measuring range (oxidizing and inert atmosphere). Simulations carried out in accordance with both kinetic models showed a very similar course of the *E_a_* curves, which confirmed our assumptions, preceded by DSC tests, that along with the degree of POM processing, its thermal and thermo-oxidative degradation is facilitated. Thus, the above results confirmed the theses put forward in our previous publication [8,9].

**Figure A7:**
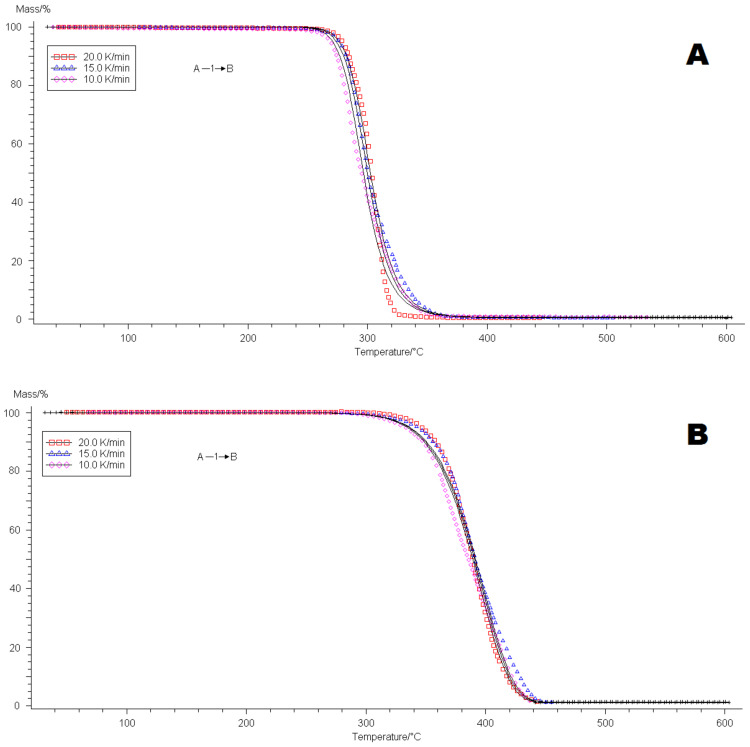
Experimental points for POM-P sample, investigated with 10, 15, and 20 °C/min heating rates, represented by simulated solid lines: (**A**) investigations carried out in oxygen atmosphere; (**B**) investigations carried out in inert atmosphere. For the simulation, the apparent activation energy was used as the function of conversion.

**Figure A8:**
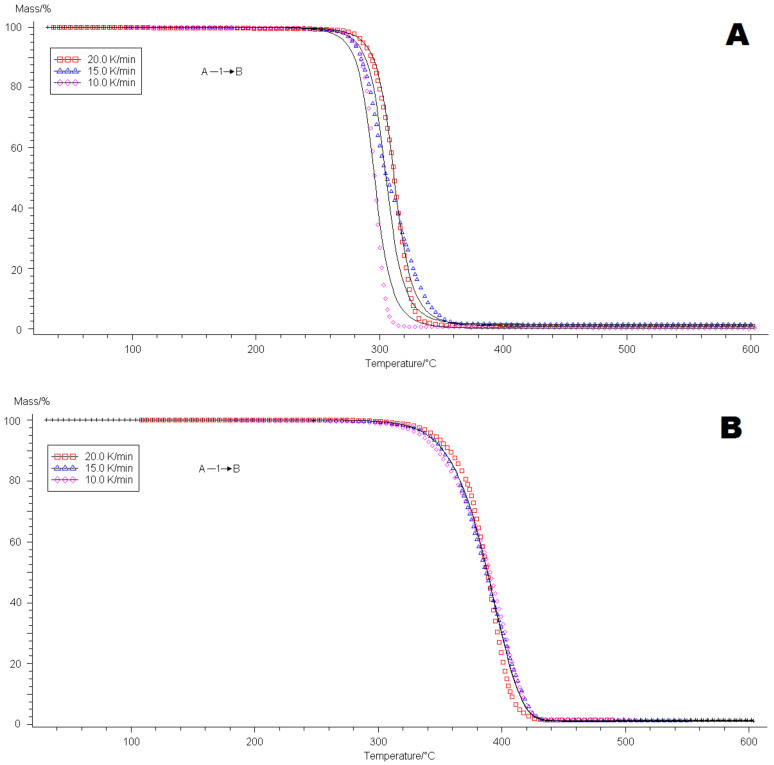
Experimental points for POM-R sample, investigated with 10, 15, and 20 °C/min heating rates, represented by simulated solid lines: (**A**) investigations carried out in oxygen atmosphere; (**B**) investigations carried out in inert atmosphere. For the simulation, the apparent activation energy was used as the function of conversion.

**Figure A9:**
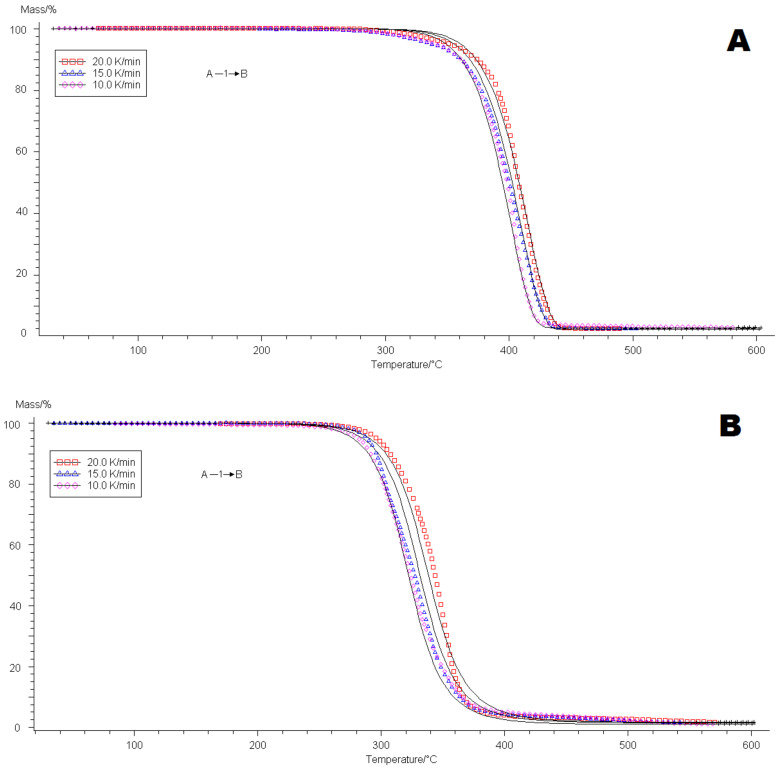
Experimental points for POM-O sample, investigated with 10, 15, and 20 °C/min heating rates, represented by simulated solid lines: (**A**) investigations carried out in oxygen atmosphere; (**B**) investigations carried out in inert atmosphere. For the simulation, the apparent activation energy was used as the function of conversion.

Knowing the values of the apparent activation energy changes as a function of partial mass loss, theoretical curves were fitted to the experimental data (F-Test) for individual samples (Appendix B: Figure A7, Figure A8 and Figure A9). In the case of all tested types of polyacetals, each of the 16 possible variants was checked in a single-step reaction (linear regression), as shown in Table 1. Then, knowing the six best linear regressions, parallel and subsequent reactions consisting of two stages in each of the 16 possible variants of X-Y (nonlinear regression) were checked as kinetic models, where X is the symbol of the first reaction, and Y is the symbol of the second reaction. The choice of X as the first reaction was dictated by the results of the six best fits using the linear regression method. It should be noted here that the calculations for the model of follow-up reactions take much longer than for parallel or competitive reactions, and the obtained results can be very similar in a statistical sense [15,16,21,22,23,24]. Table 5 shows the results of the six best matches in terms of the F-test indicator for measurements made in an oxidizing and inert atmosphere. 

In all the discussed cases of polyacetals, the best fit of the curves to the experimental data was obtained using the mechanism of single-stage or independent reactions. However, for samples decomposing under oxidative conditions, the best fit was obtained for the Cn B model (reaction of nth order with autocatalysis by product B). These results are still in good agreement with isoconversion studies for POM based on the Friedman method, where the autocatalytic nature of the POM degradation process was suggested [18,19,34]. Generally, autocatalysis occurs when the products catalyze the reaction; reactants are regenerated during a reaction. During POM degradation, the evolved formaldehyde autocatalyzes the decomposition process of POM. Autocatalysis occurs when the products catalyze the reaction; this occurs when the reactants are regenerated during a reaction in what is called branching. The reactants will eventually be consumed, and the reaction will enter a termination stage where it will cease [34]. On the other hand, for samples that decompose under neutral conditions, the best fit was obtained for the An model (n-dimensional nucleation according to Avrami-Erofeev). The kinetic parameters describing these models are listed in sequence for the POM-P, POM-R, and POM-O samples in Table 6. 

**Figure 9:**
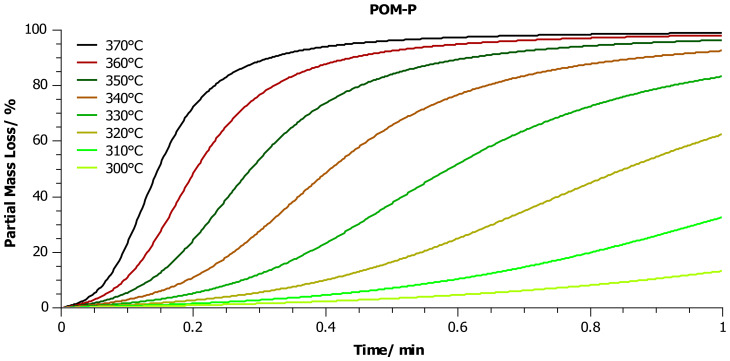
Prediction of the partial mass loss during heating in the temperature range 300–370 °C in an oxygen atmosphere. The simulation was performed for the kinetic model of the POM-P sample, which was characterized by the best fit to the experimental results (Table 5).

**Figure 10:**
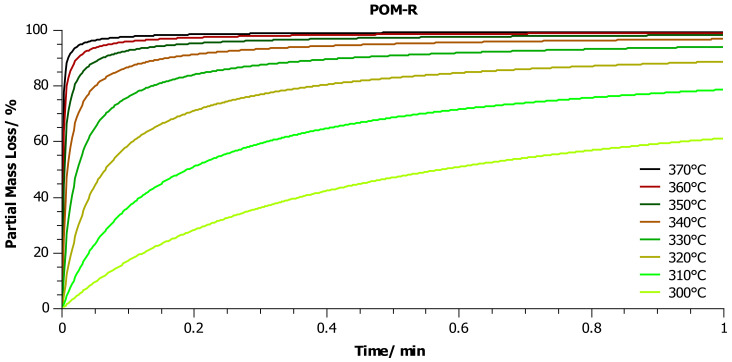
Prediction of the partial mass loss during heating in the temperature range 300–370 °C in an oxygen atmosphere. The simulation was performed for the kinetic model of the POM-R sample, which was characterized by the best fit to the experimental results (Table 5).

**Figure 11:**
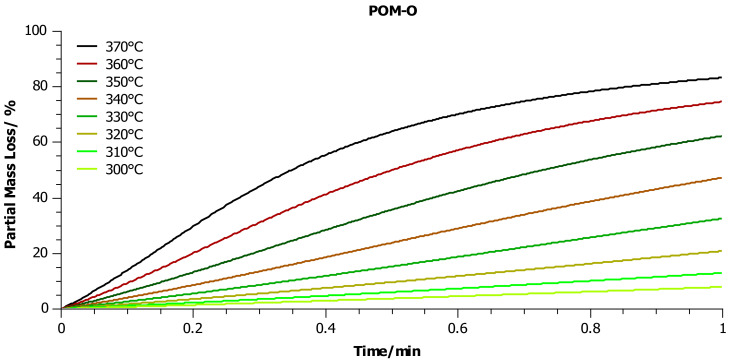
Prediction of the partial mass loss during heating in the temperature range 300–370 °C in an oxygen atmosphere. The simulation was performed for the kinetic model of the POM-O sample, which was characterized by the best fit to the experimental results (Table 5).

In a further step, thanks to the known kinetic parameters determined for individual kinetic models presented in Table 6, simulations were performed to predict the mass loss of each sample vs. time, in the temperature range from 300 to 370 °C (Figure 9, Figure 10 and Figure 11) in an oxidizing environment. As we mentioned above, the temperature range was dictated by the fact that in our previous publications [8,9] we suggested that the thermal-oxidative degradation of POM could be performed at a temperature as low as 350 °C.

As can be seen in Figure 9, Figure 10 and Figure 11, the change in the partial mass loss as a function of time showed a power character in the whole or partial temperature range. For the POM-P sample, this change was complete, and after 1 min of heating in an oxidizing atmosphere at 300 °C, about 10% of the sample was lost, and almost 99% at 360 °C. It is noteworthy that the rate of mass loss in this temperature range varied drastically, and it is anticipated that the total mass loss could be obtained after 50 s at 370 °C (Figure 9).

A slightly different trend was observed in the case of the POM-R sample. As expected, the reaction rate of POM-R decomposition should be much higher than the rate of POM-P degradation. This is illustrated, inter alia, by the degree of predicted weight loss after 1 min for individual temperatures. The complete decomposition of POM-R should take 15 s at 370 °C (Figure 10). 

On the other hand, the degradation of POM-O did not occur completely, despite the simulation carried out under comparable temperature and time conditions. The reason for this could be the presence in the sample of systems stabilizing/slowing down the decomposition reactions at elevated temperatures added during the forming of the high-temperature product, which we also found using other analytical methods in our previous publication [8,9]. The kinetic analysis showed that the addition of commercial stabilizers did not change the reaction mechanism, but only slowed it down, causing the formation of some kind of residues after the decomposition process.

## 5. Conclusions

In this article, we continued our discussions on the thermal and thermo-oxidative degradation of POM samples with different life histories. Throughout the entire study cycle of DSC measurements, the samples showed an endothermic effect. All DSC curves of the heat flow as a function of temperature for the heating process in the temperature range of 20–200 °C were characterized by the dominant phenomenon of energy absorption by the system. The calculations showed that material recycling slightly increased the degree of crystallinity in relation to the unprocessed sample. On the other hand, multiple processing and long-term use reduced the degree of crystallinity to 43%. This meant that both the processing and use of the POM detail facilitates the melting of the material, i.e., the easy transition from a glassy to a plastic state because less energy must be supplied to the system to reach the transition point between these states. Additionally, the unprocessed sample was more susceptible to temperatures above 370 °C than the repeatedly processed sample (POM-R).

The POM-P sample, without any thermal history, was characterized by a higher energy barrier, which had to be crossed in order to induce degradation, leading to its decomposition both under oxidizing and neutral conditions. On the other hand, in the case of the recycled sample (POM-R), the nature of changes in apparent activation energy was dictated by the conditions for conducting thermogravimetric measurements. The applied calculation models turned out to be consistent in terms of the nature of the curve changes and similar in terms of *E_a_* in the entire range of mass loss. Multiple processing of polyacetal (POM-O) led to a significant reduction in the apparent activation energy in the entire measuring range. Friedman and OWF kinetic models showed a very similar course of the *E_a_* curves. The samples that decompose under oxidative conditions obtained the best fit for the Cn B model (reaction of nth order with autocatalysis by product B), and the samples that decompose under inert conditions for the An model (n-dimensional nucleation according to Avrami-Erofeev). The suggestion indicated in the previous considerations to carry out combustion at a temperature of at least 350 °C seems to be correct because the predictions carried out show that the optimal temperature is 370 °C for all tested materials.

## Figures and Tables

**Table 1:** The most common types of reactions and the corresponding equations of kinetic models in two equivalent notation forms [17].

Type of Reaction	Designation	*f*(*α*)
Reaction of 1st order	F1	1 − *α*
Reaction of 2nd order	F2	(1 − *α*)^2^
Reaction of nth order	Fn	(1 − *α*)*^n^*
One-dimensional diffusion	D1	1/(2*α*)
Two-dimensional diffusion	D2	[−ln(1 − *α*)]^−1^
Three-dimensional diffusion—Jander’s type	D3	1.5(1 − *α*)^2/3^[1 − (1 − *α*)^1/3^]
Three-dimensional diffusion—Ginstling-Brounstein	D4	1.5[(1 − *α*)^−1/3^ − 1]^−1^
Two-dimensional phase boundary	R2	2(1 − *α*)^1/2^
Three-dimensional phase boundary	R3	3(1 − *α*)^2/3^
Prout–Tompkins equation	B1	*α*(1 − *α*)
Expanded Prout–Tompkins equation	Bna	(1 − *α*)*^n^α^m^*
Reaction of 1st order with autocatalysis by product	C1-X	(1 − *α*)^(1 + KcatX)^
Reaction of nth order with autocatalysis by product	Cn-X	(1 − *α*)*^n^*^(1 + KcatX)^
Two-dimensional nucleation according to Avrami	A2	2(1 − *α*)[−ln(1 − *α*)]^1/2^
Three-dimensional nucleation according to Avrami	A3	3(1 − *α*)[−ln(1 − *α*)]^2/3^
n-dimensional nucleation according to Avrami-Erofeev	An	*n*(1 − *α*)[−ln(1 − *α*)]^(*n*−1)/*n*^

**Table 2:** Designations of basic types of reactions used in kinetic analysis on the example of two-stage processes.

Type of Reaction	Scheme
One-step	$A\overset{1}{\to }B$
Independent	$\begin{array}{l} A\overset{1}{\to }B\\ C\overset{2}{\to }D \end{array}$
Consecutive	$A\overset{1}{\to }B\overset{2}{\to }C$
Parallel	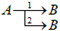
Competitive	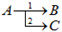

**Table 3:** Melting temperature (*T_m_*), enthalpy of fusion (Δ*H_m_*), changes in specific heat (∆*c_p_*), the degree of crystallinity (*X_c_*), and temperature of crystallization (*T_cr_*).

Sample	*T_m_*/ °C	Δ*H_m_*/J·g^−1^	∆*C_p_*/J·(g·K)^−1^	*X_c_*/ %	Tc/r °C
POM-P	167 ± 3	153.29	0.260	47	145 ± 2
POM-R	166 ± 3	161.14	0.219	49	147 ± 3
POM-O	167 ± 4	139.39	0.224	43	145 ± 2

**Table 4:** The most important thermogravimetric indicators in the graphs.

Sample	Atmosphere	*T*_5%_/°C	*T*_10%_/°C	*T*_20%_/°C	*T*_50%_/°C	*T*_max_/°C
POM-P	Inert	332 ± 7	348 ± 7	361 ± 7	386 ± 8	370 ± 8
	Oxygen	267 ± 5	274 ± 5	280 ± 6	295 ± 6	285 ± 6
POM-R	Inert	335 ± 7	348 ± 7	363 ± 7	390 ± 8	404 ± 8
	Oxygen	279 ± 6	284 ± 6	289 ± 6	295 ± 6	296 ± 6
POM-O	Inert	340 ± 7	361 ± 7	376 ± 8	398 ± 8	403 ± 8
	Oxygen	282 ± 6	291 ± 6	302 ± 6	324 ± 7	324 ± 7

**Table 5:** Six best fits of kinetic models to experimental results of POM-P, POM-R, and POM-O samples tested in oxygen and inert atmosphere (F-Test).

**POM-P**						
**#**	**Oxygen**			**Inert**		
	**Type**	**f-Act**	**F-Test**	**Type**	**f-Act**	**F-Test**
**1**	Cn B	870	1.00	An	632	1.00
**2**	Bna	869	1.03	Fn	632	1.52
**3**	Fn	869	1.03	Cn B	631	1.52
**4**	F2	871	1.08	F2	633	1.55
**5**	An	870	1.63	Bna	631	1.56
**6**	F1	871	1.99	D3	633	2.00
**POM-R**						
**#**	**Oxygen**			**Inert**		
	**Type**	**f-Act**	**Fexp**	**Type**	**f-Act**	**Fexp**
**1**	Cn B	1028	1.00	An	674	1.00
**2**	Bna	1028	1.02	D3	675	3.36
**3**	A2	1039	1.22	D4	675	4.17
**4**	C1 B	1029	1.26	F2	675	4.28
**5**	A3	1029	1.41	Fn	674	4.28
**6**	Fn	1030	1.97	Cn B	673	4.28
**POM-O**						
**#**	**Oxygen**			**Inert**		
	**Type**	**f-Act**	**Fexp**	**Type**	**f-Act**	**Fexp**
**1**	Cn B	934	1.00	An	855	1.00
**2**	Bna	934	1.03	Cn B	854	1.00
**3**	Fn	935	1.14	Bna	854	1.01
**4**	F2	936	1.19	Fn	855	1.01
**5**	F1	936	1.35	C1 B	855	1.03
**6**	An	935	1.35	R3	856	1.05

**Table 6:** Kinetic parameters determined by non-linear regression for thermal and thermo-oxidative degradation of POM-P, POM-R, and POM-O.

Factor	POM-P		POM-R		POM-O	
	Oxygen	Inert	Oxygen	Inert	Oxygen	Inert
log *A*_1_/s^−1^	26.54	30.42	6.92	10.81	8.28	12.16
*E*_1_/kJ/mol	307.08	366.89	111.53	172.33	122.95	183.95
Stand. dev. n_1_	0.41	0.26	0.23	0.07	0.35	0.19
Correlation coefficient	0.9998	0.9997	0.9998	0.9993	0.9997	0.9994

## Data Availability

Data is contained within the article.

## References

[1] Leszczyńska A., Pielichowski K., Majka T.M., Pielichowski K. (2010). The synthesis and investigation of structure–properties relationship in polyoxymethylene (POM)/montmorillonite (MMT) nanocomposites. Modern Polymeric Materials for Environmental Applications.

[2] Pielichowska K., Król K., Majka T.M. (2016). Polyoxymethylene-copolymer based composites with PEG-grafted hydroxyapatite with improved thermal stability. Thermochim. Acta.

[3] Pielichowska K. (2016). The influence of polyoxymethylene molar mass on the oxidative thermal degradation of its nanocomposites with hydroxyapatite. J. Therm. Anal. Calorim..

[4] Pielichowska K. (2015). Thermooxidative degradation of polyoxymethylene homo- and copolymer nanocomposites with hydroxyapatite: Kinetic and thermoanalytical study. Thermochim. Acta.

[5] Ramirez N.V., Sánchez-Soto M., Illescas S. (2011). Enhancement of POM thermooxidation resistance through POSS nanoparticles. Polym. Compos..

[6] Yang F., Li H., Cai L., Lan F., Xiang M. (2009). Degradation and Stabilization of Co-POM. Polym. Technol. Eng..

[7] Lüftl S., Archodoulaki V.-M., Seidler S. (2006). Thermal-oxidative induced degradation behaviour of polyoxymethylene (POM) copolymer detected by TGA/MS. Polym. Degrad. Stab..

[8] Berkowicz G., Majka T.M., Żukowski W. (2020). The pyrolysis and combustion of polyoxymethylene in a fluidised bed with the possibility of incorporating CO_2_. Energy Convers. Manag..

[9] Żukowski W., Berkowicz G., Majka T.M. (2020). Dataset on flue gas composition during pyrolysis of polyoxymethylene in a fluidised bed with the possibility of incorporating CO_2_. Data Brief.

[10] Burnham A.K., Braun R.L. (1999). Global Kinetic Analysis of Complex Materials. Energy Fuels.

[11] Moukhina E. (2012). Determination of kinetic mechanisms for reactions measured with thermoanalytical instruments. J. Therm. Anal. Calorim..

[12] Budrugeac P. (2001). The evaluation of the non-isothermal kinetic parameters of thethermal and thermo-oxidative degradation of polymers andpolymeric materials: Its use and abuse. Polym. Degrad. Stab..

[13] Opfermann J., Blumm J., Emmerich W.-D. (1998). Simulation of the sintering behavior of a ceramic green body using advanced thermokinetic analysis. Thermochim. Acta.

[14] Opfermann J. (2000). Kinetic Analysis Using Multivariate Non-linear Regression. I. Basic concepts. J. Therm. Anal. Calorim..

[15] Opfermann J., Kaisersberger E. (1992). An advantageous variant of the Ozawa-Flynn-Wall analysis. Thermochim. Acta.

[16] Herrera M., Matuscheka G., Kettrup A. (2001). Main products and kinetics of the thermal degradation of polyamides. Chemosphere.

[17] How Fast Are Chemical Reactions?. https://d2brmtk65c6tyc.cloudfront.net/fileadmin/www.netzsch-thermal-analysis.com/Microsites/Kinetics_Neo/Docs/Kinetics_NEO_en_web_2018-09-06.pdf?1558356264.

[18] Friedman H.L. (2007). Kinetics of thermal degradation of char-forming plastics from thermogravimetry. Application to a phenolic plastic. J. Polym. Sci. Part C Polym. Symp..

[19] Doyle C.D. (1962). Estimating isothermal life from thermogravimetric data. J. Appl. Polym. Sci..

[20] Ozawa T. (1965). A New Method of Analyzing Thermogravimetric Data. Bull. Chem. Soc. Jpn..

[21] Flynn J.H., Wall L.A. (1966). A quick, direct method for the determination of activation energy from thermogravimetric data. J. Polym. Sci. Part B Polym. Lett..

[22] Chrissafis K. (2009). Kinetics of thermal degradation of polymers. Complementary use of isoconversional and model-fitting methods. J. Therm. Anal. Calorim..

[23] Arshad M.A., Maaroufi A.K. (2017). Recent Progress in Kinetics of Thermal Degradation Mechanisms in Polymer Composites. MOJ Polym. Sci..

[24] Al-Salem S.M. (2019). Kinetic Studies Related to Polymer Degradation and Stability. Plastics to Energy: Fuel, Chemicals, and Sustainability Implications.

[25] Lomakin S.M., Novokshonova L.A., Brevnov P.N., Shchegolikhin A.N. (2007). Thermal properties of polyethylene/montmorillonite nanocomposites prepared by intercalative polymerization. J. Mater. Sci..

[26] Pérez-Maqueda L.A., Sánchez-Jiménez P.E., Perejón A., García-Garrido C., Criado J.M., Benítez-Guerrero M. (2014). Scission kinetic model for the prediction of polymer pyrolysis curves from chain structure. Polym. Test..

[27] Navarro R., Torre L., Kenny J., Jiménez A. (2003). Thermal degradation of recycled polypropylene toughened with elastomers. Polym. Degrad. Stab..

[28] Bordère S., Rouquerol F., Estienne J., Floreancig A. (1990). Kinetical possibilities of controlled transformation Rate Thermal Analysis (CRTA). J. Therm. Anal. Calorim..

[29] Van Krevelen D.W. (1997). Crystallization and recrystalization. Properties of Polymers.

[30] Katoh Y., Okamoto M. (2009). Crystallization controlled by layered silicates in nylon 6–clay nano-composite. Polymer.

[31] Zhao R.R. (2005). Melt Blowing Polyoxymethylene Copolymer. J. Eng. Fibers Fabr..

[32] Pielichowska K. (2012). The influence of molecular weight on the properties of polyacetal/hydroxyapatite nanocomposites. Part 1. Microstructural analysis and phase transition studies. J. Polym. Res..

[33] Hihara L.H., Adler R.P.L., Latanision R.M. (2019). Environmental Degradation of Advanced and Traditional Engineering Materials.

[34] Khawam A., Flanagan D.R. (2006). Solid-State Kinetic Models: Basics and Mathematical Fundamentals. J. Phys. Chem. B.

